# An Eco-Benign Biomimetic Approach for the Synthesis of Ni/ZnO Nanocomposite: Photocatalytic and Antioxidant Activities

**DOI:** 10.3390/molecules28041705

**Published:** 2023-02-10

**Authors:** Munirah Sulaiman Othman Alhar, Dost Muhammad, Kamran Tahir, Magdi E. A. Zaki, Muniba Urooj, Sadia Nazir, Karma Albalawi, Hamza S. Al-Shehri, Ebraheem Abdu Musad Saleh, Afaq Ullah Khan

**Affiliations:** 1 Department of Chemistry, College of Science, University of Ha’il, Ha’il 81451, Saudi Arabia; 2 Institute of Chemical Sciences, Gomal University, Dera Ismail Khan 29050, Pakistan; 3 Department of Chemistry, Faculty of Science, Imam Mohammad Ibn Saud Islamic University, Riyadh 11623, Saudi Arabia; 4 Department of Chemistry, COMSATS University Islamabad (CUI), Abbottabad Campus, Abbottabad 22060, Pakistan; 5 Department of Chemistry, Faculty of Science, University of Tabuk, Tabuk 71491, Saudi Arabia; 6 Chemistry Division, King Khalid Military Academy, SANG, Riyadh 11495, Saudi Arabia; 7 Chemistry Department, College of Arts & Science, Prince Sattam Bin Abdulaziz University, Wadi Al-Dawasir 18371, Saudi Arabia; 8 School of Chemistry and Chemical Engineering, Jiangsu University, 301 Xuefu Road, Zhenjiang 212013, China

**Keywords:** Ni/ZnO nanocomposite, photoinhibition of bacteria, hemolytic activity, antioxidant activity, photocatalytic activity

## Abstract

With the increasing demand for wastewater treatment and multidrug resistance among pathogens, it was necessary to develop an efficient catalyst with enhanced photocatalytic and antibacterial applications. The present study proposes a facile and green strategy for synthesizing zinc oxide (ZnO) decorated nickel (Ni) nanomaterials. The synthesized Ni/ZnO nanocomposite displays a high crystallinity and spherical morphology, which was systematically characterized by XRD, SEM, FT-IR, UV-visible spectroscopy, EDX, HRTEM, and XPS techniques. In addition, the bacteriological tests indicated that Ni/ZnO nanocomposite exhibits potent antibacterial activity against human pathogens, i.e., *Pseudomonas aeruginosa* (*P. aeruginosa*), *Staphylococcus aureus* (*S. aureus*), and *Escherichia coli* (*E. coli)*. The inhibition zone observed in light and dark conditions for *E. coli* was 16 (±0.3) mm and 8 (±0.4) mm, respectively, which confirms the high efficacy of the nanocomposite in the presence of light compared to dark conditions. The detailed inhibition mechanism of said bacterium and damage were also studied through fluorescence spectroscopy and SEM analysis, respectively. Evaluation of antioxidant activity based on free radical scavenging activity revealed that the Ni/ZnO nanocomposite effectively scavenges DPPH. In the photocatalytic performance, the Ni/ZnO nanocomposite exhibited a remarkable degradation ability under the optimized condition, which was attributed to their controllable size, high surface area, and exceptional morphology. Good selectivity, high photodegradation, and antibacterial activities and satisfactory hemolytic behavior of the as-prepared nanocomposite make them able to become a potential candidate for superior biological performance and environmental remediation.

## 1. Introduction

Nowadays, the quality of water is damaging for aquatic life because it is getting contaminated by the mixing of industrial waste having toxic chemicals in the form of dyes [1]. According to the researchers, the fabrication of metal oxide-based composites could have been used in the photodegradation of dye and antibacterial action against harmful microorganisms [2]. The shape, particle size, crystallinity, and porosity of these nanomaterials are all distinct physicochemical features [3,4,5]. Zinc oxide (ZnO), one of the oxides, is frequently used in photocatalytic, catalytic electronic, optical, pharmacological, magnetic, and electrocatalytic applications in both thin films and powder form [6]. Based on distinctive characteristics, ZnO is widely used in cosmeceuticals, gas sensing, laser technology, drug delivery, chemical manufacture, catalysis, and antibacterial applications [7,8,9,10,11,12,13,14].

Moreover, the ZnO NPs show antibacterial action against many pathogens, including *P. aeruginosa*, *S. aureus*, *B. subtilis*, and E. coli [15,16]. In nonclinical and clinical fields, nanoparticles can be used with antibiotics and anti-inflammation medicines to boost antibacterial activity against pathogenic microbes without causing antibiotic resistance [17]. Moreover, the biomedical applications of ZnO NPs and microparticles (MPs) have been expanded to include antimicrobial medications or medical apparatuses, implants, and cosmetics for typical clinic usage [18,19].

Another severe problem that is worsening is the release of industrial effluents, i.e., organic dyes, into water bodies [20]. In general, dyes are regarded as well-known organic pollutants since they are employed in the textile and paper industries and negatively influence human health and the environment [21]. As the dye molecules are present at ppm levels, they are challenging to see. Their continued addition causes significant toxicity in aquatic flora, fauna, humans, and the overall ecosystem [22,23]. Nanomaterials are widely used to degrade hazardous dyes to compensate for these drawbacks efficiently. For this purpose, a different group employed modified and unmodified ZnO nanomaterials used as a photocatalyst for degrading organic dyes. Generally, the doped ZnO nanomaterials demonstrate higher photocatalytic activity than pure ZnO nanoparticles [24] because it is believed that doping of metal into ZnO could increase the photocatalytic efficiency by lowering the recombination of electrons and holes [25].

Among them, hybrid nanomaterials have recently attracted a lot of attention in nanotechnology since they can change the properties of individual particles as well as provide new and improved capabilities [26]. Due to their interaction or superior qualities to their components, hybrid nanomaterials, made up of two or more diverse functional compositions, have established a key role in numerous basic applications [27,28,29]. Munir et al. have reported that NiFe_2_O_4_/ZnO demonstrates significant photocatalytic and antibacterial activities compared to the pure ZnO nanoparticles [30].

In the present work, we have successfully synthesized Ni/ZnO nanocomposites through an ammonia evaporation and co-precipitation method by using sodium hydroxide as a solvent and specific precursor. The presence of nickel on the surface of ZnO could improve the photocatalytic properties of the nanocomposite. Moreover, the as-synthesized nanomaterials showed outstanding antibacterial activity in the presence of visible light irradiation. Additionally, the efficiency of Ni/ZnO nanocomposites against MB was also permissible.

## 2. Results and Discussions

### 2.1. Optical Study

**Figure 1:**
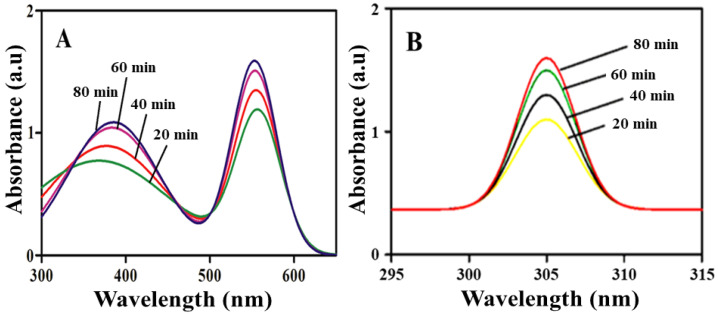
UV-visible absorbance spectra recorded for Ni/ZnO nanocomposite (**A**) and ZnO (**B**) at different time intervals.

UV-visible absorption spectroscopy has become a standard tool for determining the optical characteristics of nanomaterials. Figure 1 shows the Ni/ZnO nanocomposite absorption peaks at around 305 nm, which are caused by surface plasmon resonance excitation. The size and shape of the nanomaterial generated determine the surface plasmon resonance peak, as is well-known. Within 80 min, stable Ni/ZnO nanocomposites were produced in this investigation. After every 20 min, the production of Ni/ZnO nanocomposite was investigated, and the intensity of peaks grew with time confirming the reduction of metal ions into nanomaterials. Similar results were obtained in the Ni/ZnO nanocomposite where two peaks appeared. One peak appeared at 322 nm, representing ZnO, while the other appeared at 534 nm, corresponding to Ni. The reaction was observed up to 80 min. The gradual increase in peaks intensity was observed with the passage of time, which means more and more nanocomposite was synthesized with time.

### 2.2. XRD Analysis

**Figure 2:**
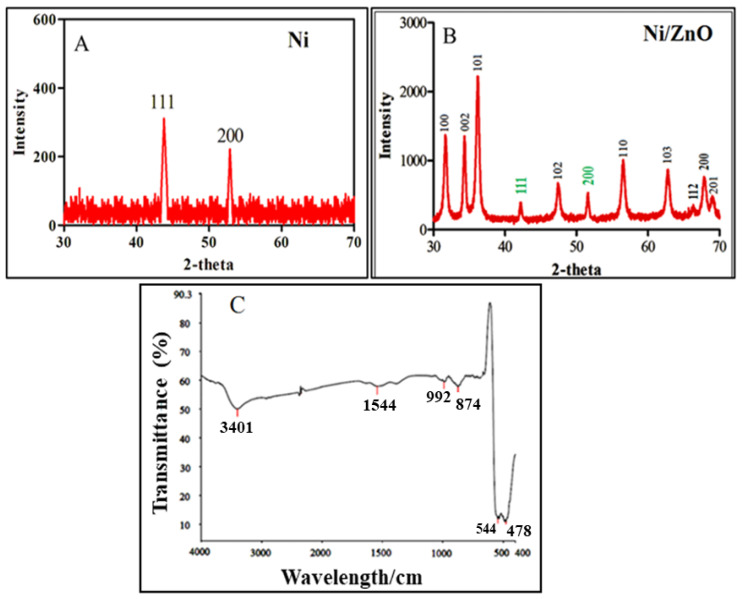
(**A**) Powder XRD pattern of the crystalline Ni nanoparticles and (**B**) Ni/ZnO nanocomposite. (**C**) FT-IR spectra of Ni/ZnO nanocomposite.

XRD is a very important analytical technique that gives information about crystal structure. Figure 2A represents the XRD study of Ni nanoparticles. Here, the two strong peaks originated at 44.2 and 53.7 that confirm the face centered cubic structure of Ni nanoparticles. Figure 2B illustrates the XRD pattern of the Ni/ZnO nanocomposite. In Ni/ZnO spectra, the remarked diffraction peaks at 31.9, 34.8, 36.4, 47.5, 56.6, 63.1, 67.0, 68.2, and 69.2° with (100), (002), (101), (102), (110), (103), (112), (200), and (201), pattern are correspond to JCPDS No. 36-1451 [31]. While two additional diffraction peaks appear at 44.5 and 51.7 in Ni/ZnO spectra are attributed to the (111) and (200) crystal planes of Ni metal (JCPDS No. 04-0850) [32]. The broad peaks obtained for synthesized nanomaterials without any additional peak confirms the purity and high crystallinity of the Ni/ZnO nanocomposite. The crystalline size of the particles can be calculated by using the Scherer equation.

Dc=kλ/βCosθ

where β is the width of the observed diffraction peak at its half maximum intensity (FWHM); K is the shape factor, whose value is equal to 0.9; and λ is X-ray wavelength (Cu Kα radiations equal to 0.154 nm). The crystalline size of Ni/ZnO nanocomposites is obtained to be approximately 21 nm.

### 2.3. FTIR Analysis

FT-IR analysis confirms the possible number of functional groups present in the sample, as displayed in Figure 2C. The spectrum is intricate due to certain precursors, solvents, and sample preparation without calcination. Six major peaks originated at 3401, 1544, 992, 874, 544, and 478 cm^−1^ indicating the presence of different functional groups. The well-observed broad peak at 3401 cm^−1^ corresponds to the -OH stretching vibration [33]. The peak at 1544 cm^−1^ belongs to the -OH bending vibration. The two peaks at 992 cm^−1^ and 874 cm^−1^ were assigns to the Ni-O-Ni and Zn-O-Zn stretching vibrations, respectively. In addition, two major peaks at 544 and 478 cm^−1^ have also appeared, which associate to the N = O and Zn-O stretching vibrations, respectively.

### 2.4. HRTEM Analysis and Particle Size and Morphology

**Figure 3:**
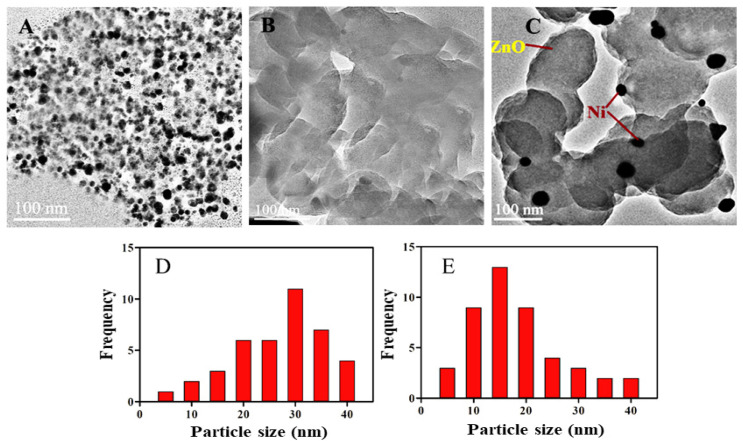
(**A**) Typical HRTEM graphs of (**A**) Ni, (**B**) ZnO, (**C**) Ni/ZnO nanocomposite, and (**D**,**E**) particle size distribution of Ni both individual and in composite with ZnO, respectively.

The surface morphology, shape, and size of the Ni-doped ZnO composite was investigated by using HRTEM. Figure 3A–C represent individual Ni, ZnO, and nanocomposite Ni/ZnO, respectively. In the case of individual Ni nanoparticles, aggregation was observed among the particles; as a result, the size was increased (average size more than 30 nm; Figure 3D). However, in the case of the Ni/ZnO nanocomposite, the size of Ni was well-controlled with a high quality of dispersion. The average size was observed to have 13 nm (Figure 3E). Additionally, Ni nanoparticles have small-sized, spherical shapes and are uniformly distributed over ZnO, which designates that ZnO is the best support and has the aptitude to produce highly dispersed and small-sized Ni-NPs.

### 2.5. SEM and EDX Analysis

**Figure 4:**
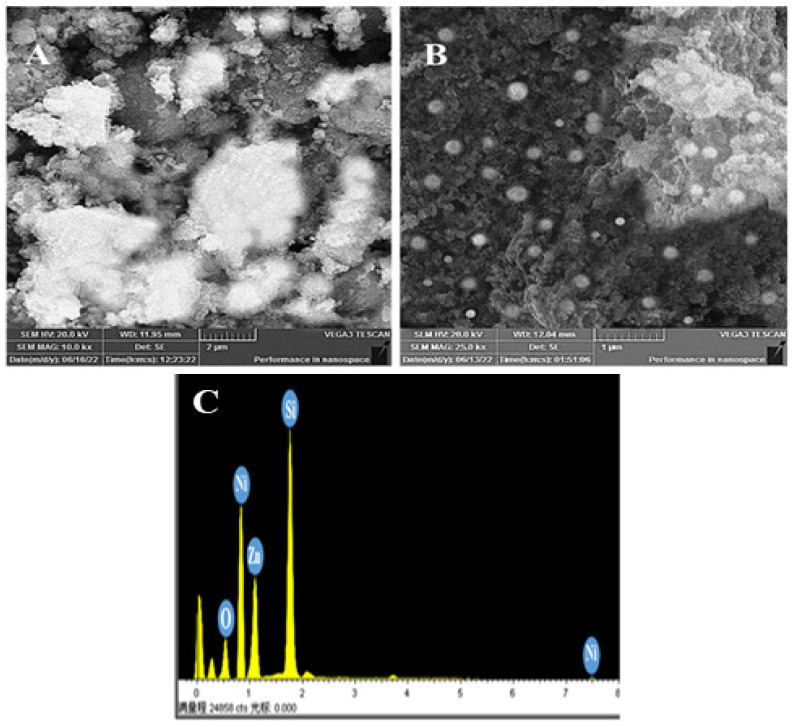
Typical SEM graphs of (**A**) ZnO, (**B**) Ni/ZnO, and (**C**) EDX spectral analysis of Ni/ZnO nanocomposite.

The image of the synthesized nanocomposite is given in Figure 4A,B. Figure 4A shows ZnO nanomaterials that are present in sheet form with a porous surface. On the other hand, Figure 4B shows an SEM analysis of Ni/ZnO where the Ni nanoparticles are present on the ZnO surface with a spherical control shape and are highly distributed over the rough surface of ZnO.

A EDX was used to confirm the elemental composition of the Ni/ZnO nanocomposite. As seen in Figure 4C, the EDX spectrum indisputably supports the production of Ni-coated ZnO nanomaterials. It is evident from the result that well-observed peaks appeared for Ni, Zn, and O, which confirm the formation of Ni over the surface of ZnO nanoparticles.

### 2.6. Photocatalytic Activity

**Figure 5:**
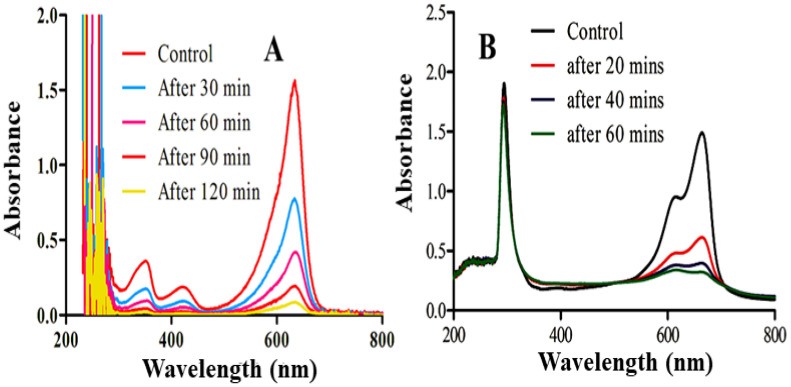
UV-visible absorption spectra during the photocatalytic degradation of MB in the presence of (**A**) ZnO and (**B**) the Ni/ZnO nanocomposite under visible light irradiation.

Considering the environmental perspective, photocatalysis has become the most applicable and expanded field at the broader range in the nanoscale regime. Ideally, Ni/ZnO photocatalysts are considered the most promising candidate due to their low cost and lack of toxicity in the degradation of organic pollutants. From previous studies, it has been found that industries play an adverse role in the contamination of drinking water by discharging a great variety of toxic pollutants, which in turn affect the living organisms. As the dying process concerns, it causes the release of approximately 10–50% of polluted products into the water streams disturbing the natural environment [34,35]. Among different investigated dyes, MB comes out as a well-known coloring thiazine pollutant. The highly stable MB acts as a model dye in the evaluation of the photocatalytic efficiency of the Ni/ZnO nanocomposite. Two significant peaks for MB were displayed at 665 nm and 614 nm and the photodegradation process was carried out. The results clearly indicated 100% degradation of MB is carried out by the application of Ni/ZnO nanocomposites only in 60 min when irradiated with UV-visible light, as illustrated in Figure 5A. There are several reasons which are responsible for the maximum photocatalytic efficiency of Ni-doped ZnO nanomaterials, which are discussed as follows: accordingly, the Ni/ZnO nanocomposite has shown more capability of light absorption, and Ni nanoparticles are highly dispersed on the surface of spherical shaped ZnO, which results in an increase of surface area with more active sites. Furthermore, a closer observation of Figure 5B shows that individual ZnO presents less activity.

Interestingly, Photocatalytic activity depends on several features, such as crystal structure, size, and morphology of the particle [36]. It is presumably established that certain important factors, such as light absorption, charge transportation, and separation on the catalyst surface, are used to determine the mechanism of photodegradation efficiency [37]. The smaller size and charge transfer from Ni to ZnO, which significantly decreased the recombination of electrons and increased the photodegradation efficiency of the nanocomposite, were responsible for the rapid photodegradation of MB.

#### 2.6.1. Factors Affecting the Photodegradation of MB

There are several factors, such as catalyst concentration, initial dye concentration, pH of solution, etc., which affect the photodegradation of MB. In order to elevate the degradation process of MB, it is very important to analyze which factor has a great effect and which value of the said factors gives successful performance.

#### 2.6.2. Effect of Catalyst Dosage

**Figure 6:**
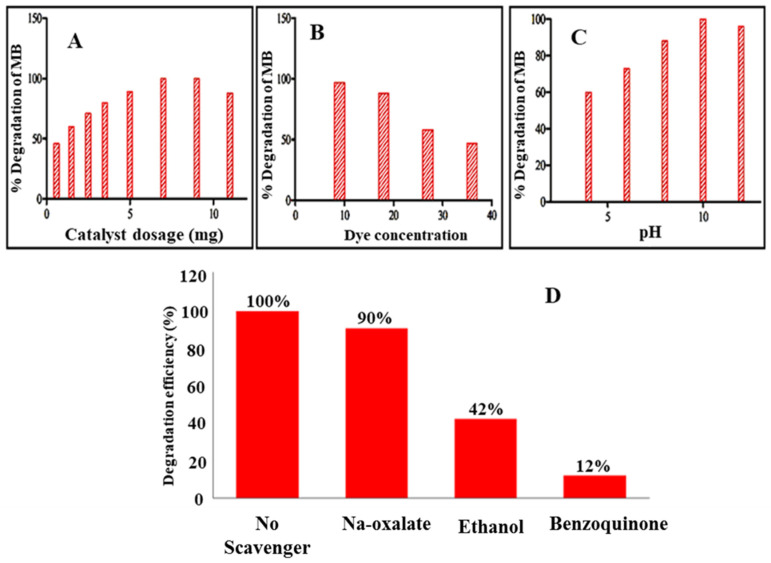
(**A**) Effect of catalyst dosage (**B**) effect of dye concentration, (**C**) effect of pH on the photodegradation of MB in the presence of Ni/ZnO nanocomposite, (**D**) Scavenger test.

The effect of various concentrations of the Ni/ZnO nanocomposite (0.6, 1.5, 2.5, 3.5, 5, 7, 9, and 11 mg) was studied as illustrated in Figure 6A. Visible light (300 V bulb) was used as a light source for 5 min by adding 0.6, 1.5, 2.5, 3.5, 5, 7, 9, and 11 mg of Ni/ZnO in 80 mL (16 mg/L) of MB solution. The results designated that by increasing the amount of the Ni/ZnO nanocomposite from 0.6, 1.5, 2.5, 3.5, 5, 7, and 9 mg, an increase in the photodegradation of MB was observed: 46%, 60%, 71%, 80%, 89%, and 100%. It was also observed that increasing the concentration of the Ni/ZnO nanocomposite (11 mg) decreases the photodegradation of MB. It is because the active sites and ROS production is increased by boosting the concentrations of the Ni/ZnO nanocomposite to an optimum amount (9 mg). However, if the concentration of Ni/ZnO increases from the optimum (9 mg) to an excess amount (11 mg), a decrease in the photodegradation of MB (88%) was observed. For instance, increasing the photocatalyst concentration causes turbidity/muddiness in a solution, in which light cannot scatter. Hence the active sites and the production of ROS are decreased. Thus we can conclude that an increase in the concentration of photocatalyst to an optimum value will increase the photodegradation of MB but exceeding this value will decrease the photodegradation.

#### 2.6.3. Effect of Initial Dye Concentration

Different initial concentrations of MB dye have an influence on the photodegradation efficiency of Ni/ZnO nanocomposites. In this experiment, 30 mg of Ni/ZnO and 80 mL of water with dye concentrations of 9, 18, 27, and 36 mg/L were utilized (Figure 6B). The colloidal solution was irradiated for 5 min. Initially, a considerable amount of dye degradation (97%) was observed due to the standard conversion of H2O2 to hydroxyl radicals. These •OH radicals have a concise life span, i.e., less than nanoseconds, and immediately react when they are produced. The photodegradation efficiency of Ni/ZnO decreases gradually (88%, 58%, and 47%) by increasing the MB dye concentration. It is due to the production of •OH radicals on the surface of Ni/ZnO nanocomposites as MB molecules cover the active sites of nickel-doped zinc oxide.

#### 2.6.4. Effect of Solution pH

Moreover, the pH is an important parameter of a solution. It can directly affect the crystal size, surface charge, and aggregation of catalyst particles. The •OH radicals significantly affect dye degradation, and these radical’s concentration mostly depends on the pH of a solution. In this experiment, the effect of pH on the Ni/ZnO nanocomposite’s catalytic effectiveness was investigated by either raising or lowering the pH from 9.3.

The addition of NaOH and HCl adjusted the solution’s pH. To check the influence of the pH, the solutions have a pH range of 4–11. The solution was irradiated for 60 min with (9 mg/L) dye concentration. The result indicates that 60%, 70%, 80%, 100%, and 96% MB were degraded at pH values of 4, 6, 8, 10, and 11, respectively (Figure 6C). This means that with an increase in pH up to an optimum value (10), the catalyst shows high photocatalytic efficiency and the maximum amount (100%) of MB was degraded. While in case of pH higher than 10, a reduction in the photodegradation efficiency of catalysts was recorded.

Consequently, as demonstrated previously in the photocatalysis mechanism, at an acidic pH the surface of the catalyst is protonated by the excessive generation of H^+^ ions. In case of MB, which exists in the cationic form in water, the repulsion results between dye molecule and catalyst nanocomposites. At alkaline pH, more ˉOH radicals are generated. Such type of radicals are mainly responsible for oxidation and reduction reactions, which ultimately results in the complete degradation of hazardous dye in the presence of UV-visible light irradiation.

### 2.7. Scavenging Test

The reactive oxygen species played a vital role in the degradation of organic dyes. An experiment was performed to authenticate the ROS production in the presence of Ni/ZnO nanocomposites, liable for the degradation of MB. Several scavengers, such as sodium oxalate (Na_2_C_2_O_4_), ethanol (C_2_H_5_OH), and benzoquinone (C_6_H_4_O_2_) were applied to investigate the production of ROS (O_2_•^−^, •OH, and h+) as shown in Figure 6D. For this purpose, 10 mg of Ni/ZnO nanomaterials were used as photocatalyst for the deprivation of MB. The Ni/ZnO nanocomposites and scavengers, i.e., sodium oxalate, ethanol, and benzoquinone, were added into the aqueous solution of MB (2 Mm) to scavenge the reactive oxygen species i.e., h+, •OH, and O2•^−^. It was inspected that MB’s maximum (100%) degradation was observed in the absence of scavengers. All the ROS were produced after visible light irradiation without the presence of the scavengers. A conspicuous effect has been shown by benzoquinone and ethanol compared to sodium oxalate. It designates that O2•^−^ and •OH are the more active species compare to h+ for the photodegradation of MB in the presence of Ni/ZnO photocatalysts.

### 2.8. Antibacterial Test

**Figure 7:**
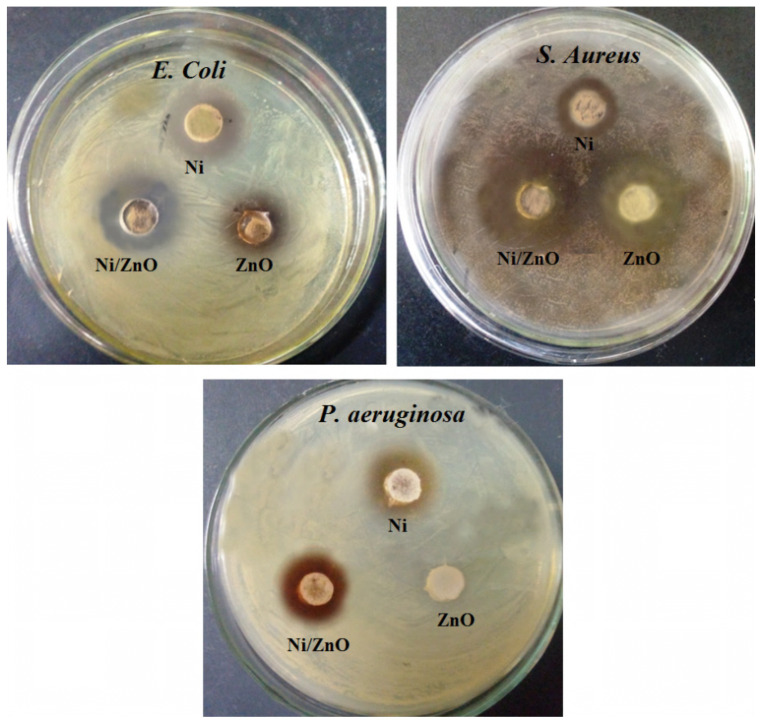
Antibacterial activity of individual Ni, ZnO, and Ni/ZnO nanomaterial against the selected bacteria.

In this research work, the antibacterial activity of Ni/ZnO nanocomposites was analyzed against test bacteria, that is, *E. coli*, P. aeruginosa, and *S. aureus*. The activity was judged both in the light and dark against the test bacteria, which are responsible for the creation of infections in humans, including urinary tract infections, bacteremia, cholangitis, etc. [38]. Table 1 summarized the comparison of different synthesized materials, which exhibits that exposing Ni/ZnO nanocomposites to visible light increases their antibacterial effectiveness against particular bacteria and inhibits their growth. The antibacterial activity for the Ni/ZnO nanocomposite, when carried out in the presence of visible light, depicts surprisingly efficient activity concerning the activity performed in the dark, as shown in Table 1 and Figure 7. The table represents the inhibition diameter for irradiated Ni/ZnO, which was 16 (±0.3), 19 (±0.4), and 23 (±0.5) mm, which corresponds to *E. coli*, P. aeruginosa, and *S. aureus*, respectively. On the other hand, the inhibition diameter in the dark was 8 (±0.4), 10 (±0.4), and 14 (±0.5) mm, respectively. Furthermore, the degradation turnover (dTON), which is employed to assess contaminant degradation in catalytic activities, can be used to describe the comparison of catalysts in terms of numeric values regardless of the concentration of the contaminant and the amount of the catalyst. The dTON was calculated by the following equation [39]:

$$\mathrm{dTON}=\frac{\left[\mathrm{Mi}\right]-\left[\mathrm{Mf}\right]}{t\times [\mathrm{Cat}.]}$$

where Mi and Mf denote the initial and final concentrations of the bacteria after treatment (mmol or μmol), respectively; t represents time (h); and Cat. denotes the catalyst amount (g). Finally, the dTON of 413, 392, 311, and 298 μmol h^−1^ g^−1^_Cat_. were obtained for irradiated Ni/ZnO, dark Ni/ZnO, ZnO, and Ni samples, respectively.

**Figure 8:**
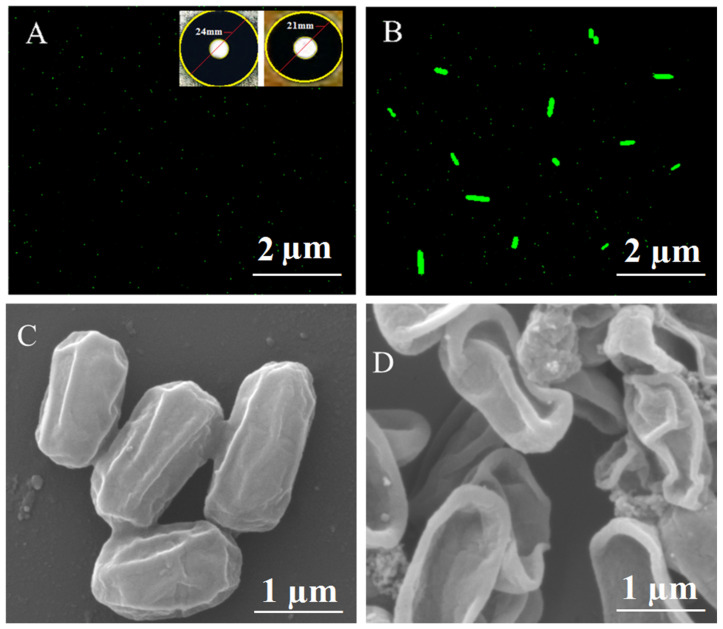
ROS examination in *E. coli* in the (**A**) absence (**B**) presence of the Ni/ZnO nanocomposite; (**C**) SEM examination of E. coli before addition of the Ni/ZnO nanocomposite; and (**D**) after the addition of the Ni/ZnO nanocomposite.

The inhibition diameter depicts more significant antibacterial activity, which is carried out for the Ni/ZnO nanocomposite in the presence of visible light. The phenomenon of *E. coli* cell structural degradation was also observed using SEM in the presence and absence of the Ni/ZnO nanocomposite, as illustrated in Figure 8C, D. According to the results of the Ni/ZnO nanocomposite, E. coli’s shape and structure changed significantly when exposed to light.

### 2.9. MIC of Ni/ZnO Nanocomposite

The MIC of the Ni/ZnO nanocomposite for each test species was determined in triplicates using a serial dilution approach. Initially, we took a gradient concentration sequence of the Ni/ZnO nanocomposite (20 to 70 µg/mL). The MIC against *E. coli* and P. aeruginosa was found to be 30 µg/mL, while against *S. aureus* it was 20 µg/mL, as shown in Table 2.

### 2.10. Determination of ROS

Different studies show that metallic nanoagents are significant candidates for ROS production [40]. In the microbial cell, excited electrons from prepared nanomaterials produce ROS species, such as •OH, ‾•O_2_, and H_2_O_2_. These reactive species cause oxidative stress in the cell breaking intracellular biomolecules, such as protein, DNA, and the cell membrane. In the presence of reactive species, 2, 7-dichlorofluorescein-diacetate oxidizes to dichlorofluoroscein resulting in green fluorescence when excited at 488 nm, as shown in Figure 8A,B [41]. Intracellular fluorescence was seen in treated bacterial samples exposed to Ni/ZnO nanocomposites indicating that our prepared Ni/ZnO nanocomposite generates reactive oxygen species.

#### Proposed Mechanism for Antimicrobial Test

The exact mechanism of the antibacterial activity delivered by the nanoparticles was not clear due to one different reason. In recent work, a great deal of effort has tried to explain the exact mechanism of the antibacterial activity depicted by the green synthesized Ni/ZnO nanocomposite in the presence of visible light. According to the recent mechanism, it has been concluded that when visible light strikes the surface, it ejects electrons available in the ground state, which in turn reacts with O_2_ and H_2_O_2_ and leads to the formation of superoxides. The electronic holes, in association with water molecules, generate OH radicals. Finally, superoxides and OH radicals combine with bacterial membranes, eventually leading to cell death.

From the literature survey and in this research project, it has been clear that credit of antibacterial activity has been achieved by the ZnO, which aids in the generation of ROS [42]. These ROS produced as a result of the excitation of electrons from the nanomaterials, which in turn promotes the generation of ROS and causes the destruction of the *E. coli* cell. Moreover, the oxidation of 2, 7-dichorofluorescin-diacetate dye into dichlorofluoroscein also takes due to ROS and green fluorescence appearing upon excitation at 488 nm. The surprising results clarify the significant role of the Ni/ZnO nanocomposite in the creation of ROS, while interaction with *E. coli* in turn leads to the leakage of the cytoplasm of bacterial cells as shown in Figure 8D [43].

### 2.11. Hemolytic Activity

To determine the biocompatibility with RBCs at several specified concentrations, i.e., 12.5, 25, 50, 75, 100, and 125 µg, the hemolytic activity of the Ni/ZnO nanocomposite was evaluated. As distinct phenolic compounds served as capping agents, it was noted that the Ni/ZnO nanocomposite exhibited no hemolytic activity. The capping agents, such as condensed tannins, epicatechin, and procyanidin A2, were derived from fresh litchi skin and pericarps as indicated in Table 3. These phenolic substances significantly increase hemolysis resistance while lowering the possibility of free radical-induced oxidative damage to the erythrocytes [44].

### 2.12. Antioxidant Activity

**Figure 9:**
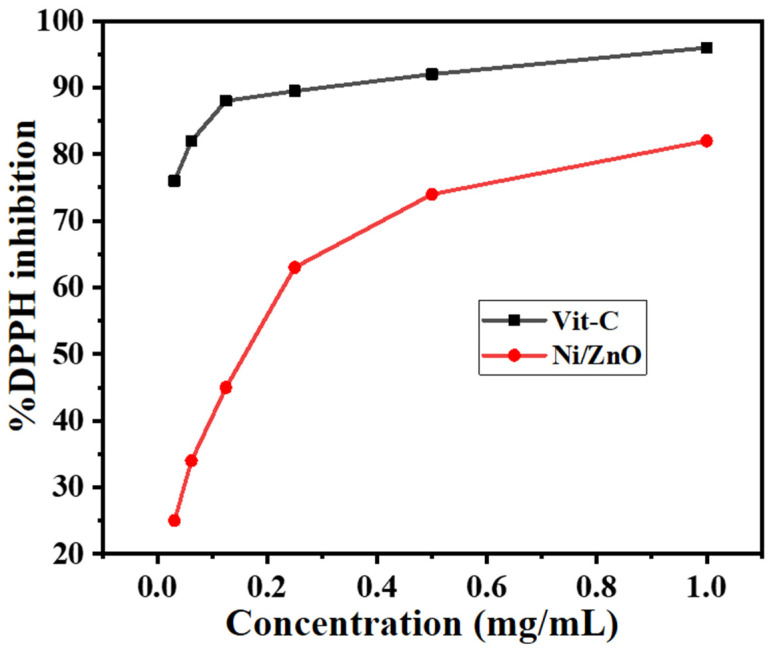
Antioxidant activity of Ni/ZnO nanocomposite against DPPH.

The antioxidant test of the Ni/ZnO nanocomposite was assessed by utilizing vitamin C as a reference and DPPH conversion from radical to a stable form. According to the findings in Figure 9, nearly all radicals were stabilized, and the radical conversion efficiency of the Ni/ZnO nanocomposite increased with concentration. When the Ni/ZnO nanocomposite concentration was 1 mg/mL, however, the inhibition of DPPH may increase to a maximum of 82%. Apart from the Ni/ZnO nanocomposites high chemical activity, the high capacity of the Ni/ZnO nanocomposite to deactivate these free radicles could be attributed to the dispersibility of nanoparticles through the media due to their tiny particle size. The oxygen-releasing antioxidant may boost cells ability to multiply, migrate, grow, disseminate, and accelerate the healing process [45]. Therefore, Ni/ZnO nanocomposite exhibits a moderate antioxidant activity.

## 3. Experimental

### 3.1. Synthesis of ZnO

Firstly, 1.148 g zinc sulfate (ZnSO_4_·7H_2_O), 99.5%, Sigma Aldrich, St. Louis, MO, USA) was dispersed in 40 mL distilled water and sonicated for 10 min. In another beaker, 0.48 g of sodium hydroxide (NaOH, 98.44%, Sigma Aldrich, St. Louis, MO, USA) was dissolved in 30 mL of water and this solution was added dropwise to the first soluble zinc sulfate solution. As a result, the addition of NaOH solution with continuous stirring formed zinc hydroxide. The mixture was filtered through a suction pump using Watt man filter paper, and white precipitates of zinc hydroxide were obtained. To eliminate the undesired contaminants, the zinc hydroxide precipitates were washed three times with DDW and ethanol twice to remove unwanted particles. After that, they were dried in a microwave oven at 60 °C for six hours. After drying, calcination occurred at 400 °C for 4 h to obtain zinc oxide.

### 3.2. Post-Synthesis of Ni/ZnO Nanocomposite

The preparation of the Ni-doped ZnO nanocomposite began with the dissolution of 0.1 g of Nickel(II) acetylacetonate (Ni(acac)_2_, 90, Sigma Aldrich, St. Louis, MO, USA) in 20 mm of distilled water in a single beaker. The solution was sonicated for 10–15 min. In a separate beaker containing 80 mL of distilled water, 2 g of the ZnO nanoparticles that had already been synthesized were dissolved. The nickel acetate solution was added to the zinc oxide solution one drop at a time while the mixture was continuously stirred. Ammonia solution (30%) was added to keep the pH of the mixture in the basic range. The stirring is continued for the next 2 h. The appearance of light-green color confirms the formation of the Ni-doped ZnO nanocomposite. The mixture was filtered through a centrifuge (3000 rpm, 10 min), washed with distilled water to remove the impurities, and then dried in a microwave oven at 60 °C for 24 h.

### 3.3. Photocatalytic Activity Test

Methylene blue (MB) was selected as the pollutant to study the photocatalytic activity of biosynthesized Ni/ZnO nanocomposite under visible light illumination. For this, 4 mg of the Ni/ZnO nanocomposite was poured into 25 mL of the proposed dye solution and stirred for 35 min to achieve absorption desorption equilibrium. Then, the solution was exposed to visible light irradiation (300 W, xenon lamp with 420 nm cut filter) and continuously stirred. After an equal interval of time, 3 mL of the suspension were taken through a pipette and centrifuged to remove the Ni/ZnO composite particles, and then absorption of MB was monitored by using spectrophotometer (Shimadzu 2450).

### 3.4. Antibacterial Activity of Ni/ZnO Nanocomposite

Using the agar well diffusion method, the antibacterial activity of the Ni/ZnO nanocomposite was evaluated against three bacterial strains, namely E. coli, S. aureus, and P. aeruginosa [46]. The bacterial strains were cultivated in broth media and kept at 37 °C until they were transferred to Petri dishes using a sterile glass rod. Using a sterile cork borer, 6 mm-wide holes were drilled into nutrition agar (Merck, Darmstadt, Germany) plates. Laterally, a suspension of 1 mg/mL of Ni/ZnO nanocomposite were prepared in double distilled water. Then, 50 µL suspension of Ni/ZnO nanocomposite were carefully poured into the holes by using a micropipette and kept the plates for 80 min under visible light irradiation. Finally, the plates were incubated for 18 to 20 h at 37 °C to determine the zone of inhibition.

### 3.5. Reactive Oxygen Species (ROS) Test

The formation of reactive oxygen species, which is measured by the presence of an important 2, 7-dichlorodihydrofluorescein diacetate dye (ThermoFisher, Waltham, MA, USA), was the cause of the nanocomposites antibacterial activity. The E. coli strain and a precise quantity of the Ni/ZnO nanocomposite were added to the experiment and incubated for 3 h at 300 rpm. The bacterial cell solution was collected after incubation and combined for 60 min with 1 mL of 15 mM 2, 7-dichlorodihydrofluorescein diacetate dye. The mixture was rinsed with PBS solution to get the dye from the surface of the bacterial cells. The fluorescence was measured at two specified wavelengths of 488 nm, which corresponds to the phenomenon of excitation and emission, respectively.

### 3.6. Minimum Inhibitory Concentration (MIC)

Agar well dilution process was implemented to evaluate the antibacterial activity of the green synthesized Ni/ZnO nanocomposite. The activity was analyzed on the basis of MIC value. The experiment was conducted with various concentrations of Ni/ZnO nanocomposite. Approximately 1 mL solution of nanocomposite of various concentrations was mixed with 1 mL of each bacterial solution. After dissolution, the mixture was introduced into Petri dishes maintained at 37 °C in an incubator for 20 h. In contrast, the test tube that did not contain any concentration of nanocomposite was regarded as the experiment’s negative control. The concentration of Ni/ZnO nanocomposite utilized in the experiment ranges from 20 to 70 µg/mL. The experiment was repeated thrice to get an accurate MIC value of the nanocomposite.

### 3.7. Hemolytic Activity

The nanocomposite pellets were utilized to assess the hemolytic property by measuring the hemoglobin released from the red blood cells. After selecting the blood of a male Wister albino mouse, it is then placed in sterile heparin vacutainers for further testing. After this, the supernatant was removed, and the remaining pellet was washed with a buffer solution of PBS at pH 7.4 and placed in a separate tube with different concentrations of 12.5, 25, 50, 75, 100, and 125 µg in PBS solution. The volume of the solution was made up to 1 mL. The negative and positive controls were considered as RBCs in PBS and 1% Triton-X 100 solution in the experiment. The reaction mixture was introduced in a shaker with incubation conditions of 37 °C for 1 h and was further centrifuged at 15 rpm for a period of 10 min. The percentage of hemolytic activity was measured at 540 nm compared to the blank solution and calculated by the previously reported equation [40].

### 3.8. Antioxidant Activity

As previously reported, the antioxidant capabilities of the produced Ni/ZnO nanocomposite were studied using the 2, 2-diphenyl-1-picrylhydrazyl (DPPH, Sigma Aldrich, St. Louis, MO, USA) radical [31]. In this step, a various concentration i.e., 0.031, 0.062, 0.125, 0.25, 0.5, and 1 mg/mL of Ni/ZnO nanocomposite were diluted in methanol and mixed separately with 0.5 mL of 1 mM DPPH. These were then placed in the dark for an incubation period of 30 min. Ascorbic acid was used as a reference in the experiment. In the negative control trial, DPPH solvent was employed without any samples. Whereas, using a UV-visible spectrometer (UV-2400; Shimadzu, Japan), maximum absorption for DPPH was detected at 517 nm; a progressive drop in absorption was observed when DPPH interacted with antioxidant. Finally, the antioxidant activity level was calculated by the formula [47]:

$$\mathrm{Percent}\;\mathrm{Inhibition}=\frac{A-B_{1}}{A}\times 100$$

where A is the absorbance of the control and B_1_ is the tested sample, respectively.

## 4. Conclusions

In this research, we have employed a facile and eco-friendly method for the production of the Ni-doped ZnO nanocomposite, which was used for photocatalytic and biological applications. The as-prepared nanomaterials possess a nearly small size, desired morphology, and spherical shape with partial aggregation. In addition, the current study unfolds that synthesized nanomaterial has excellent light-induced inhibitory efficiency against both Gram-positive and Gram-negative bacteria and exhibits no hemolytic activity. Moreover, it was found that the Ni/ZnO nanocomposite possesses excellent catalytic performance against MB degradation. Further, the high effectiveness of as-synthesized material against different microbes is observed due to ROS production that could destroy the microorganism growth. A vital benefit of the Ni/ZnO nanocomposite is its remarkable ability to scavenge the highly stable radical DPPH. This study opened a new perspective to design nanomaterials that are multiplicative and have great potential in environmental remediation and biorelated applications.

## Figures and Tables

**Table 1:** Antibacterial activity of Ni, ZnO, and Ni/ZnO nanocomposite against different pathogenic bacteria.

Bacterial Strains	Zone of Inhibition (mm)				
	Ni	ZnO	Irradiated Ni/ZnO	Dark Ni/ZnO	Positive Control
*E. coli*	11 ± 0.4	13 ± 0.3	16 ± 0.3	8 ± 0.4	Zero inhibition
*P. aeruginosa*	9 ± 0.3	14 ± 0.4	19 ± 0.4	10 ± 0.4	Zero inhibition
*S. aureus*	9 ± 0.2	5 ± 0.3	23 ± 0.5	14 ± 0.5	Zero inhibition

**Table 2:** MIC of Ni/ZnO nanocomposite.

Bacteria	Ni/ZnO Nanocomposite (µg/mL)						
	Control	70	60	50	40	30	20
*E. coli*	**+**	**−**	**−**	**−**	**−**	**−**	**+**
*P. aeruginosa*	**+**	**−**	**−**	**−**	**−**	**−**	**+**
*S. aureus*	**+**	**−**	**−**	**−**	**−**	**−**	**−**

+, growth of bacteria; −, inhibition of bacteria

**Table 3:** Hemolytic efficiency of Ni/ZnO nanocomposite.

Sample (*n* = 3) (μg)	Hemolytic Activity (%) (OD_540 nm_)
Control 1% Triton X-100 Ni/ZnO (12.5) Ni/ZnO (25) Ni/ZnO (50) Ni/ZnO (75) Ni/ZnO (100) Ni/ZnO (125)	1.18 ± 0.11 99.9 ± 0.3 1.23 ± 0.8 1.28 ± 0.10 1.28 ± 0.11 1.30 ± 0.10 1.31 ± 0.9 1.33 ± 0.10

OD_540_ nm is optical density at 540 nm.

## Data Availability

Not applicable.
